# A Retrospective, Single-Center Study of Technical-Procedural Factors Affecting Radiation Dose During Prostatic Artery Embolization

**DOI:** 10.7759/cureus.27728

**Published:** 2022-08-06

**Authors:** Hippocrates Moschouris, Konstantinos Stamatiou, Nektarios Spanomanolis, Anastasios Vasilopoulos, Spiros Tzamarias, Katerina Malagari

**Affiliations:** 1 Department of Radiology, Tzaneio General Hospital, Piraeus, GRC; 2 Department of Urology, Tzaneio General Hospital, Piraeus, GRC; 3 Second Department of Radiology, Attikon University Hospital, Athens, GRC

**Keywords:** dose area product, computed tomography angiography, radiation dose, cone-beam computed tomography, roadmap, digital subtraction angiography, prostatic artery embolization

## Abstract

Introduction

This study aims to evaluate the effect of technical-procedural factors on radiation dose during prostatic artery embolization (PAE).

Methods

This was a single-center, retrospective study of 59 patients with benign prostatic hyperplasia (BPH) who underwent prostatic artery embolization from March 2020 to September 2021. Computed tomography angiography (CTA) was performed for vascular planning prior to PAE in all patients. The effect of the following techniques on the dose area product (DAP) of PAE was evaluated: application of low-dose protocol (LDP) for digital subtraction angiography (DSA), reduction of oblique projections by performing PAE of at least one pelvic side utilizing anteroposterior projections only (AP-PAE), utilization of “roadmap” technique instead of DSA for the delineation of pelvic arterial anatomy (RDMP-PAE), and cone-beam CT (CBCT). The impact of the patient’s body mass index (BMI) on DAP was also calculated. The effective dose (ED) of PAE and pre-PAE CTA was calculated from DAP and from dose length products, respectively, using appropriate conversion factors.

Results

For the entire study population (n = 59), the mean DAP of PAE was 16,424.7 ± 8,019 μGy‧m^2^. On simple regression analysis, LDP, AP-PAE, and RDMP-PAE significantly contributed to DAP reduction during PAE (30% (p = 0.004), 26.7% (p = 0.013), and 31.2% (p = 0.004), respectively). On multiple regression, LDP and AP-PAE maintained their significant effect (p = 0.002 and p = 0.006, respectively). CBCT was associated with a not statistically significant increase in DAP (10.1%) (p = 0.555). The ED of CTA represented 21.2% ± 10.6% of the ED of PAE.

Conclusion

Of the four studied factors, LDP, AP-PAE, and RDMP-PAE proved to be relatively simple and widely available techniques that could limit radiation exposure of both the operators and the patients during PAE. The contribution of planning CTA to the overall radiation exposure of patients undergoing PAE appears to be not negligible.

## Introduction

Prostatic artery embolization (PAE) has gained increasing acceptance as a minimally invasive treatment of symptomatic benign prostatic hyperplasia (BPH), combining satisfactory clinical efficacy with an excellent safety profile [1]. One of the few aspects of PAE that have raised skepticism is radiation exposure (both for patients and for operators), which results from long fluoroscopy times, numerous angiographic runs, and steep oblique projections [2,3]. In response, techniques to limit radiation dose during PAE have been developed. Ιntraprocedural cone-beam computed tomography (CBCT) has been used by some groups as an aid for the identification and selection of prostatic artery, with a consequent reduction in fluoroscopy time, angiographic runs, and radiation exposure [4,5]; however, these benefits have been challenged by others [6]. A low-dose angiographic protocol (combined with image processing to compensate for the lower image quality associated with this protocol) was found to result in a 74% reduction in radiation dose received by the main operator during PAE compared to the standard protocol [7]. However, the utilization of such a low-dose protocol has not been reported in large PAE studies. PAE with anteroposterior (AP) imaging only and intentionally unilateral PAE have also been proposed as options to limit radiation dose [8,9], but they have not been adequately validated yet. Further research is therefore required to extensively test existing and newer approaches for the reduction of radiation exposure during PAE and identify the most efficient and practical of these solutions, which could be incorporated into the standard PAE procedure.

The present work reflects the recent experience of a single center in the utilization of several techniques that could contribute to the reduction of radiation dose during PAE. The primary objective of this study is to review these techniques and assess their benefits in terms of radiation dose reduction. The secondary objective is to assess additional factors associated with the total radiation exposure during PAE and report on the efficacy and safety of PAE in the studied patient cohort.

## Materials and methods

Patients

Patients with symptomatic BPH who were treated with PAE in a single tertiary center from March 2020 to September 2021 with a follow-up time of at least two months were retrospectively reviewed. The inclusion criteria for PAE were as follows: age ≥ 50 years, prostate enlargement (sonographically calculated prostate volume of more than 35 mL), moderate-to-severe lower urinary tract symptoms (International Prostate Symptom Score (IPSS) > 18), failure of medical treatment (5α-reductase inhibitors or selective α1-blockers administered for at least six months), and urinary retention managed with indwelling bladder catheter with at least three failed attempts of catheter removal prior to PAE. The exclusion criteria for PAE were as follows: previous surgical or interventional prostate treatments, urinary tract infection, prostate or bladder cancer, neurogenic bladder, bladder diverticula larger than 3 cm, bladder stones, and contraindications to angiography [10]. Computed tomography angiography (CTA) was performed for treatment planning for all patients prior to PAE (Table 1).

**Table 1:** Basic features/parameters of pelvic CTA (arterial phase only) utilized for this study. CTA: computed tomography angiography; IV: intravenous; ROI: region of interest; HU: Hounsfield units

Feature or parameter	Details or value
Equipment	64-row scanner (Optima CT 660, GE Healthcare, WI, USA)
Kilovoltage	120 kV
Milliamperage	Automatic
Matrix	512 × 512 pixels
Collimation	64 × 0.625 mm
Slice thickness	1.25 mm
Pitch	0.984:1
Iodinated contrast	1.5 mL/kg of patient’s weight, 350 mg iodine/mL
IV injection rate	4.5-5 mL/s
Bolus triggering	ROI just above aortic bifurcation, with a threshold of 250 HU

All procedures were performed with the same angiographic unit (Axiom Artis Zee, Siemens Healthineers, Erlangen, Germany) and by the same first operator (five years of previous experience in PAE).

Written informed consent was obtained from all patients prior to PAE. The study was approved by the formal research ethics review committee of the institution of the study (approval number: 1950/2021).

Technique

The basic PAE techniques for the present study were as follows. After local anesthesia, vascular access was obtained via the common femoral artery with the Seldinger technique. A 4 or 5 French (F), 65-80 cm, reverse curve catheter (Cobra 1, Simmons 1, MOT, Merit Medical, USA, or Boston Scientific, USA) was utilized for the selection of the ipsilateral internal iliac artery (IIA) and for the cross-over maneuver. If the catheterization of the contralateral IIA could not be accomplished with the same catheter, it was exchanged for a “vertebral” or “Bern” one. Digital subtraction angiography (DSA) of the IIAs (with the standard “Body” protocol of the equipment) was performed with manual injection of 10-20 mL of iodinated contrast through the angiographic catheter on anteroposterior and ipsilateral anterior oblique (30^o^-40^o^, often with additional caudal-cranial angulation up to 10^o^) projections to document arterial anatomy and identify the origin of the prostatic arteries. Oblique roadmaps were produced from the corresponding oblique DSAs (with the “DSA-Roadmap” function of the equipment) and were used to guide the catheterization of the prostatic artery; this was accomplished with a microcatheter (1.7-2 F, Velout, Tellus, or Stridesmooth Asahi Intecc Co., Japan) and a double-angled 0.016-inch microguidewire (Meister-Asahi). Intra-arterial nitroglycerin was administered through the microcatheter for vasodilation, and DSAs were acquired with manual injection of 2-4 mL of contrast to study in detail the prostatic artery and its branches or anastomoses. Embolization was performed until complete flow stasis with microspheres (Embosphere, Merit Medical, USA) (diameters: 100-500 microns).

The following modifications of the aforementioned basic techniques were applied in subgroups of patients, and the effect of these modifications on radiation dose was evaluated.

Low-Dose Protocol Instead of the Standard “Body” Protocol for DSA

**Figure 1:**
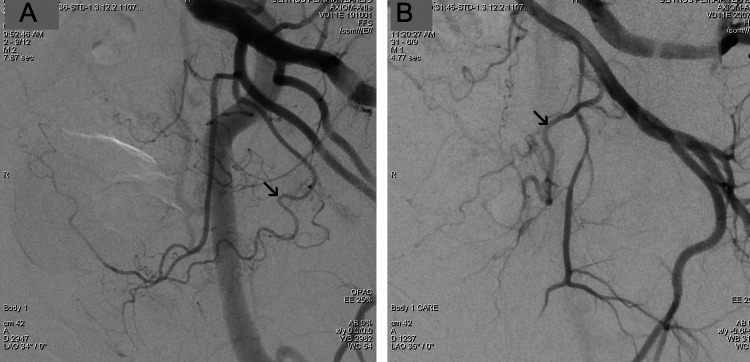
Digital subtraction angiography (DSA) images (A,B) acquired with similar tube angulation and no magnification from two patients in the study show the pelvic arterial anatomy relevant to PAE. The standard DSA protocol (“Body”) was applied in (A), and the low-dose DSA protocol (“Body CARE”) was applied in (B). Prostatic arteries (arrows in both images) can be easily identified in both images; however, the application of low-dose DSA protocol was associated with a significantly lower dose area product (DAP) (64.4 μGy‧ m2/frame versus 152 μGy‧m2/frame), despite the much higher body mass index (BMI) of the patient of image B (38 versus 27).

The preset LDP of the angiographic unit (Combined Applications to Reduce Exposure (CARE): Body CARE®-1 protocol, Siemens) is characterized by reduced pulse width and increased edge enhancement compared to the standard (“Body”) protocol (Figure 1).

Additional parameters for LDP and for the standard protocol are provided in Table 2.

**Table 2:** Basic parameters of the different protocols applied in the angiographic unit for this study. kV: kilovolts; ms: milliseconds; Body 1: standard DSA protocol; Body CARE 1: low-dose DSA protocol; CBCT: cone-beam computed tomography; fr: frame

Parameter	Imaging protocol			
	Body 1	Body CARE 1	Fluoroscopy	CBCT
Kilovoltage	70 kV	70 kV	73 kV	90 kV
Pulse width	80 ms	50 ms	12.5 ms	12.5 ms
Dose	3,000 μGy/fr	1,200 μGy/fr	-	0.360 μGy/fr
Edge enhance (natural)	45%	50%	20%	40%
Edge enhance (subtraction)	10%	25%	-	-
Angle	-	-	-	200°
Angulation step	-	-	-	1.5°/fr

For the purposes of this study, LDP was considered as having been successfully applied when all DSA acquisitions of a PAE procedure had been acquired with this protocol.

Reduction of Oblique Projections

Although these projections are fundamental in the delineation of the prostatic artery (particularly its origin), they are also associated with significantly higher radiation doses than anteroposterior (AP) views. With a recently described approach [8], it is possible, in cases with larger prostatic arteries and with favorable anatomy, to perform PAE utilizing only anteroposterior fluoroscopy and/or DSA (AP-PAE). In the present study, AP-PAE was considered as having been successfully applied when embolization of the prostatic artery of at least one pelvic side had been accomplished with AP projections only. It should be noticed that in all modern angiographic systems, the X-ray generator is under the table; therefore, in a supine patient, projections of AP-PAE are actually posteroanterior and not anteroposterior. However, the acronym AP (instead of PA) was preferred to avoid confusion with the acronym of the prostatic artery and to be consistent with previous references to the technique.

Utilization of Roadmap Images Instead of DSA Runs for the Demonstration of the IIA Branches and for Anatomic Orientation

In the context of this study, this approach was termed “RDMP-PAE” and entailed the acquisition of roadmaps (after the activation of the “Roadmap” function, fluoroscopy, and manual contrast injection) instead of DSAs for all contrast injections through the angiographic catheter and for both pelvic sides. The same technique was applied for some superselective injections through the microcatheter; however, after the prostatic artery had been selected, at least one DSA run per pelvic side was performed, pre- and post-embolization, to maximize image detail and ensure the identification of smaller branches and anastomoses.

Cone-Beam Computed Tomography (CBCT)

This was selectively applied to elucidate arterial anatomy when there was uncertainty on pre-procedural CTA and on intraprocedural DSA. For this purpose, “proximal” CBCT was performed with an injection of 45 mL of iodinated contrast (diluted 50% with normal saline) into the IIA, through the angiographic catheter, at a rate of 5 mL/second by a mechanical injector and with a delay of four seconds. CBCT was also performed in selected cases to confirm the presence of small but potentially hazardous anastomoses of the prostatic artery with other arterial branches (vesical, rectal, and penile). In these cases, contrast injection was performed distally through the microcatheter (“distal” CBCT) and required manual injection of 3 mL of contrast (same dilution as before) at approximately 0.5 mL/second and with a two-second delay. For both techniques, CBCT was performed using a five-second rotational scan of 200° (Table 2). The acquired images were transferred to a dedicated workstation (Leonardo, Siemens), where three-dimensional reconstructions and maximum intensity projections were produced.

Finally, the following techniques and parameters were always applied in all patients to reduce radiation exposure. All DSA runs were acquired at a rate of one frame (fr)/second. Fluoroscopy was performed at seven pulses (p)/second and was reduced to 2-4 p/second during the injection of the embolic. At this phase of the procedure, tight collimation (including only the prostatic area and the site of potential anastomoses) was also applied. No magnification was used during the DSA runs. The preset digital “zoom” function (×2) provided by the manufacturer was instead applied to the acquired images.

Assessment and recording of radiation dose

After each PAE procedure, the total dose area product (DAP) (μGy‧m^2^) was recorded by the angiographic unit and extracted for analysis. The dose length product (DLP) (in mGy‧cm ) of pre-procedural planning CTA was also recorded. For each patient, the effective dose (ED) of CTA was calculated from DLP using 0.015 mSv/(mGy‧cm) as the conversion factor [11,12]. The ED of the PAE procedure for each patient was calculated from DAP using the conversion factor for “Pelvic arterial embolization” (0.0026 mSv/(μGy‧m^2^)) [11,13].

Follow-up

Clinical and imaging (transabdominal ultrasound) follow-up was scheduled at approximately three, six, and 12 months post-PAE and then every six months. Changes (compared to baseline measurements) in IPSS, prostate volume, and post-void residual (PVR) were recorded. Clinical success was defined as a post-treatment IPSS of ≤15 points with a decrease of at least 25% from the baseline, with no need for additional treatment and (for patients with indwelling bladder catheter) as permanent catheter removal with spontaneous micturition and PVR < 100 mL.

Statistical analysis

Descriptive statistics were calculated for quantitative and qualitative data. The normality of variables was assessed using skewness, kurtosis, and Shapiro-Wilk test. The four aforementioned techniques (LDP, AP-PAE, RDMP-PAE, and CBCT) were examined as determinants of DAP with simple regression analysis in the form of independent samples t-test. The effect of body mass index (BMI) on DAP was also evaluated using a simple regression analysis. Factors with a statistically significant effect on DAP were then studied with multiple linear regression. The Kaplan-Meier method was used to calculate the clinical success rates of PAE over time. Statistical significance was defined as a p-value of <0.05.

## Results

General

A total of 66 patients underwent PAE during the study period. Seven of them were excluded (for two of them, no follow-up was available; the other five patients were excluded for reasons described below). The baseline data for the 59 patients that were eventually included in the study are provided in Table 3.

**Table 3:** Baseline demographic and clinical data of the patients (n = 59) in this study. SD: standard deviation; BMI: body mass index; PV: prostate volume; IPSS: International Prostate Symptom Score; PVR: post-void residual

Variable	Mean ± SD
Age (years)	71.5 ± 10.9
BMI	26.8 ± 2.9
PV (mL)	94.6 ± 41.2
PVR (mL)	106 ± 84
IPSS (mean ± SD)	25 ± 5

Prior to PAE, 39 patients suffered from moderate or severe lower urinary tract symptoms, while 20 had indwelling bladder catheters. Bilateral PAE was performed in 47/59 patients (79.6%), while the rest underwent unilateral PAE. For the 59 patients in the study, the mean DAP was 16,424.7 ± 8,019 μGy‧m^2^ (range: 3,127-39,416 μGy‧m^2^).

Determinants of DAP

During the first nine months of the study, PAE was performed with the standard DSA protocol (“Body” protocol, no LDP) in 24/59 patients. After a brief training and familiarization of the operators with the LDP (“Body CARE 1”) of the system and during the last nine months of the study, all PAE procedures (35/59 patients) were performed with LDP. In all these procedures, image quality was satisfactory, and no switch to the standard protocol was required. LDP resulted in significantly lower DAP values on simple regression analysis (30% reduction, p = 0.004). Both LDP and standard protocol were unintentionally applied in a separate case, which was excluded.

AP-PAE was successfully performed in 31/59 (52.5%) patients. Embolization of both pelvic sides with AP-PAE was achieved in 6/31 patients; embolization of only one pelvic side with AP-PAE was achieved in 25/31 patients (the other pelvic side in these patients was embolized with oblique views from the beginning, as per basic technique). In three other patients, AP-PAE was unsuccessful and was followed by oblique projections. These three patients were excluded from the study, although the failed AP approach in them accounted for a mean of only 3.2% of the total DAP. In 28/59 patients, PAE was performed with the basic technique (oblique projections from the beginning, no AP-PAE attempted). Compared to this group, AP-PAE achieved a 26.7% reduction in DAP (p = 0.013).

In the first 35 patients of the study, the procedure entailed the constant acquisition of DSAs for the mapping of the arterial anatomy. RDMP-PAE was applied in the last 24 patients of the study, after the observation that roadmaps (instead of DSAs) were adequate for the delineation of the main splanchnic branches of the IIA and particularly of the prostatic artery origin. Compared to the standard technique, RDMP-PAE resulted in significantly lower DAP (31.2% reduction, p = 0.004). In one separate case, DSAs were performed to clarify arterial anatomy after the RDMP technique, and this patient was excluded from the study.

CBCT was performed in 8/59 patients (nine CBCT scans in total, seven “proximal” and two “distal”). Although CBCT was associated with a 10.1% increase in DAP, the difference compared to non-CBCT cases was not statistically significant. More detailed data on the effects of LDP, AP-PAE, RDMP-PAE, and CBCT on DAP are provided in Table 4.

**Table 4:** Effect of technical factors on DAP (simple regression analysis). LDP: low-dose protocol; AP-PAE: anteroposterior prostatic artery embolization; RDMP-PAE: roadmap prostatic artery embolization; CBCT: cone-beam computed tomography; SD: standard deviation; DAP: dose area product; *: statistically significant

Technique	Number of patients	DAP (mean ± SD) (μGym^2^)	p
LDP - applied	35	13,981.5 ± 6,910.2	0.004*
LDP - not applied	24	19,987.8 ± 8,321.1	
AP-PAE - applied	31	14,004.8 ± 6,999.1	0.013*
AP-PAE - not applied	28	19,104 ± 8,338.9	
RDMP-PAE - applied	25	13,013.7 ± 6,141.3	0.004*
RDMP-PAE - not applied	34	18,932.8 ± 8,384.1	
CBCT - applied	8	18,000.4 ± 10,404.3	0.555
CBCT - not applied	51	16,177.6 ± 7,680	

Of the aforementioned determinants, LDP and AP-PAE maintained their significance in multiple regression analysis. LDP had the greatest and most statistically significant effect on DAP; the utilization of LDP in a PAE session was associated with an average reduction of DAP by 5,443 μGy‧m^2^. BMI was also an important determinant of DAP on both simple and multiple regression (Table 5).

**Table 5:** Effect of technical factors and of BMI on DAP (multiple regression analysis). LDP: low-dose protocol; AP-PAE: anteroposterior prostatic artery embolization; RDMP-PAE: roadmap prostatic artery embolization; BMI: body mass index; B: unstandardized regression coefficient; CI: confidence interval; β: standardized regression coefficient; *: statistically significant

Technique	B	95% CI for B		β	p
		Lower bound	Upper bound		
(Constant)	-9605.703	-24288,112	5076,705		0.195
RDMP-PAE	-2869.910	-6439,862	700,042	-0.178	0.113
AP-PAE	-4874.663	-8285,882	-1463,445	-0.306	0.006*
LDP	-5443.083	-8836,404	-2049,762	-0.336	0.002*
ΒΜΙ	1233.650	684,703	1782,596	0.454	<0.001*

Effective dose and contribution of CTA

The mean ED of PAE was calculated at 42.7 ± 20.8 mSv. Pre-PAE CTA was associated with a mean ED of 7.8 ± 3.3 mSv (range: 3.2-20.3 mSv). This represented 21.8% ± 11% (range: 8%-63%) of the ED of the PAE procedure. The effect of the patient’s BMI on ED of planning CTA was statistically significant (p = 0.001).

Clinical outcomes

**Figure 2:**
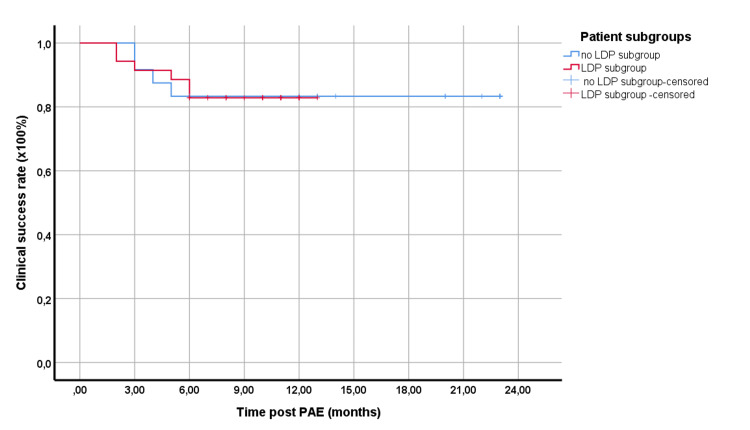
Kaplan-Meier curves showing the clinical success rates of the two main subgroups of the study. Red line: the subgroup with the application of LDP, with or without other techniques; blue line: the rest of the patients Differences were not statistically significant (p = 0.514). LDP: low-dose protocol

Follow-up time ranged from two to 23 months (mean: 12.02; median: 11 months). For the total patient cohort, the clinical success was 91.6%, 83.1%, 83.1%, and 83.1% at three, six, 12, and 18 months post-PAE, respectively. To better reflect the center’s current practice of PAE (with the application of LDP in all patients), the respective subgroup (in which LDP, with or without other techniques, was applied (n = 35)) was separately examined. The clinical success rates for this subgroup, which underwent PAE with even lower DAP (13,981.5 ± 6,910.2 μGy‧m^2^) were not significantly different from the rest (p = 0.968) (Figure 2).

Only minor complications were observed in the treated patients (acute urinary retention, n = 3, one of them in the LDP subgroup; transient, self-limiting hemospermia, n = 2, one of them in the LDP subgroup; and small groin hematoma, n = 1, of the LDP subgroup).

## Discussion

The techniques that were evaluated in this study and proved effective in reducing radiation dose during PAE are easy to apply and require equipment that is incorporated in every modern angiographic unit; moreover, they were applied in “real-life” conditions in consecutive (nonselected) PAE candidates in a tertiary center. Of note, the benefit of radiation dose reduction was combined with a satisfactory clinical success rate, comparable to those of large studies from high-volume centers [14] and with the absence of any significant complications.

LDP proved to be the easiest and most efficient technique to limit radiation dose during PAE. Although the study group included large patients, the anatomic detail provided by LDP was adequate, and no switch to the standard protocol was required. In a previous study, Andrade et al. used the same equipment and an LDP for PAE and recorded even lower DAP (11,680 μGy‧m^2^ or 74% lower than their standard protocol) [7]. However, a detailed comparison with the present results is not possible, since the aforementioned study included a small number of patients, and BMI (also an important determinant of DAP) was not reported.

AP-PAE was another effective dose-limiting technique in both simple and multiple regression analyses. As emphasized in its original description [8], AP-PAE relies heavily on preprocedural CTA and is more likely to succeed in cases with larger PAs and with favorable anatomy. Compared to this first report, in the present series, AP-PAE could be successfully applied in significantly more patients (52.5% versus 26%), perhaps as a result of the newer equipment and of increased experience; AP-PAE failed in less than 10% of the patients in whom it was attempted (three patients, eventually excluded from the study), and even in these cases, the additional dose attributed to AP-PAE was negligible. Of note, a variant of the herein adopted AP-PAE technique was utilized in a large multicenter PAE study [9] and also contributed significantly to dose limitation.

RDMP-PAE was a significant determinant of dose reduction only on simple regression analysis. However, in practice, RDMP provides adequate image quality and anatomic detail with a lower dose than a corresponding DSA run; therefore, the RDMP technique is currently used by the authors instead of DSA, prior to superselective prostatic catheterization in almost all PAE procedures. A similar approach for the reduction of angiographic runs has been proposed for uterine fibroid embolization [15].

The herein presented results regarding CBCT are at variance with those of other studies, in which CBCT was used as the standard modality for vascular planning (instead of CTA) and contributed significantly to the reduction of overall radiation exposure [4,5]. This variance is probably explained by the different role of CBCT in the present study, where CBCT was applied only in a minority of patients with particularly challenging anatomy and/or complex anastomoses; it is therefore not surprising that CBCT failed to achieve a reduction in radiation exposure in this demanding subgroup of patients who would anyway require longer fluoroscopy times and numerous DSAs. It should also be noticed that in the present study, no attempts were made to reduce radiation dose during the CBCT scans (e.g., with a low-dose CBCT program).

The mean DAP value for the entire patient cohort of this study (16,424.7 ± 8,019 μGy‧m^2^) is lower than the overall mean DAP (18,100.6 μGy‧m^2^) reported in a systematic review [2] on radiation exposure during PAE. As already noticed, the current status in the center of the present study is better reflected by the subgroup of patients who underwent PAE with LDP (with or without other techniques), and in this subgroup, an even lower mean DAP was recorded (13,981.5 ± 6,910.2 μGy‧m^2^). This is comparable to the results of a recent report (mean DAP: 14,890 ± 9,250 μGy‧m^2^) in which CBCT and a large display monitor proved valuable in limiting radiation exposure during PAE [5]. Different combinations of techniques, depending on the local availability, preferences, and expertise, may therefore be almost equally effective in radiation reduction during PAE.

Finally, although CTA is very commonly used for vascular planning prior to PAE, limited data exist regarding the contribution of this planning CTA to the total radiation exposure of patients undergoing PAE. The single-phase (arterial only) CTA protocol utilized in this study resulted in an ED that was lower than elsewhere reported [11]; nevertheless, this ED still represented a not negligible percentage of the ED of the PAE procedure (mean: 21%). Therefore, it would be worth investigating the role of low-dose techniques in planning CTA for PAE.

The limitations of the present work include its retrospective nature and the absence of direct dose measurements. Moreover, although patients with challenging arterial anatomy, stenoses, and tortuosities were treated, the impact of these factors on DAP was not specifically analyzed.

## Conclusions

LDP, AP-PAE, and RDMP-PAE are relatively simple techniques that can be applied with standard angiographic equipment to limit radiation exposure during PAE. This benefit is not associated with any compromise in the safety and efficacy of PAE. The contribution of planning CTA to the total radiation exposure of patients undergoing PAE appears to be not negligible.
